# Thermal, Mechanical, and Rheological Properties of Biocomposites Made of Poly(lactic acid) and Potato Pulp Powder

**DOI:** 10.3390/ijms20030675

**Published:** 2019-02-05

**Authors:** Maria Cristina Righetti, Patrizia Cinelli, Norma Mallegni, Carlo Andrea Massa, Simona Bronco, Andreas Stäbler, Andrea Lazzeri

**Affiliations:** 1 CNR-IPCF, National Research Council—Institute for Chemical and Physical Processes, Via Moruzzi 1, 56124 Pisa, Italy; norma.mallegni@pi.ipcf.cnr.it (N.M.); carlo.andrea.massa@pi.ipcf.cnr.it (C.A.M.); simona.bronco@pi.ipcf.cnr.it (S.B.); andrea.lazzeri@unipi.it (A.L.); 2 Department of Civil and Industrial Engineering, University of Pisa, Largo Lucio Lazzarino 1, 56122 Pisa, Italy; 3 Fraunhofer Institute for Process Engineering and Packaging IVV, Giggenhauser Straße, 35, 85354 Freising, Germany; andreas.staebler@ivv.fraunhofer.de

**Keywords:** bio-based polymers, natural fibers, biomass, biocomposites, fiber/matrix adhesion

## Abstract

The thermal, mechanical, and rheological properties of biocomposites of poly(lactic acid) (PLA) with potato pulp powder were investigated in order to (1) quantify how the addition of this filler modifies the structure of the polymeric material and (2) to obtain information on the possible miscibility and compatibility between PLA and the potato pulp. The potato pulp powder utilized is a residue of the processing for the production and extraction of starch. The study was conducted by analyzing the effect of the potato pulp concentration on the thermal, mechanical, and rheological properties of the biocomposites. The results showed that the potato pulp powder does not act as reinforcement but as filler for the PLA polymeric matrix. A progressive decrease in elastic modulus, tensile strength, and elongation at break was observed with increasing the potato pulp percentage. This moderate loss of mechanical properties, however, still meets the technical requirements indicated for the production of rigid packaging items. The incorporation of potato pulp powder to PLA offers the possibility to reduce the cost of the final products and promotes a circular economy approach for the valorization of agro-food waste biomass.

## 1. Introduction

Biodegradable bio-based polymers obtained from renewable resources represent an important alternative to petroleum-derived non-degradable polymers. For this reason, bio-based polymers—for example, poly(lactic acid) (PLA), polyhydroxylalkanoates (PHA), in particular polyhydroxylbutyrate (PHB) and its copolymers poly(hydroxylbutyrate-co-valerate) (PHBV), cellulose, and starch derived plastics, which are produced from renewable agricultural and biomass feedstock—have become a subject of crucial interest for academia and industry. Among them, PLA is the biodegradable polymer most present on the market and widely used for packaging purposes. Many properties of PLA, such as strength and stiffness, are comparable to those of traditional petroleum-based polymers. On the other hand, the drawbacks for its applications are the low toughness and the relatively low glass transition, which limits its use at a temperature above about 50 °C, because, due to its slow crystallization rate, processing of PLA generally results in amorphous products [1]. In addition, bio-based polymers are generally more expensive than conventional petroleum-derived polymers and often exhibit worse thermal and mechanical properties, which make them unacceptable for several applications.

In order to balance the cost and achieve suitable properties for different applications, a possible solution is offered by biocomposites. Biocomposites are a special class of composite materials obtained by blending natural fibers with bio-based polymers. Biocomposites represent an ecological and low-cost alternative to conventional petroleum-derived materials and, for this reason, are becoming progressively more utilized for a wide variety of uses. Natural fiber reinforced biocomposites have been reviewed in several articles [2,3,4,5,6,7].

Besides the benefit of being biodegradable and produced from renewable resources, natural fibers are also less abrasive to processing equipment than synthetic fibers like ceramic or glass fibers and traditional fillers like mica and glass, and they exhibit a lower density, which makes biocomposites lightweight and economical materials [8].

The chemical composition of the natural fibers, which varies depending on the plant from which they are derived, consists mainly of cellulose (50–70 wt%), hemicellulose (10–20 wt%), lignin (10–30 wt%), and pectin and waxes in smaller amounts [7]. The physico-mechanical properties of the natural fibers also depend on the original plants. The density, elastic modulus, tensile strength, and elongation at break are approximately in the ranges 0.8–1.5 g/cm^3^, 5–20 GPa, 200–900 MPa, and 1.5–20%, respectively [7].

The mechanical properties of a biocomposite results from both the matrix and fiber properties [9,10] and strongly depend on the matrix/fiber interface [11]. The tensile strength is more sensitive to the matrix/fiber adhesion, whereas the modulus depends, in general, on both the matrix and the fiber properties. The mass or volume fraction of the fibers, the fiber aspect ratio (length to width ratio), the fiber–matrix adhesion, and the fiber orientation are crucial factors responsible for the final properties of natural fiber reinforced composites [12]. In a composite, the matrix plays the role of transferring the applied stress to the fibers. This occurs at the interface, and therefore a good matrix/fiber adhesion is necessary. Poor adhesion often characterizes biocomposites made of hydrophobic polymers and hydrophilic natural fibers, which can lead to scarce mechanical properties due to the tendency of the fibers to form aggregates during processing. In addition, the hydrophilic nature of the lignocellulosic fibers induces absorption of moisture in an amount that can vary up to about 10%, with the result that if the fibers are not efficiently dried previously, the process ability is worsened, and the formation of porous products is thus unluckily favored. The fiber aspect ratio strongly influences the tensile modulus and the fracture properties, and a critical fiber length is necessary to develop composites with high stiffness and strength. Fibers with low aspect ratio and irregular shape behave as fillers and not as reinforcement. Thus, multiple factors influence the mechanical properties of a composite, not only the intrinsic properties of the fibers but also their shape and dimensions and their dispersion in the polymeric matrix together with the interactions that are established at the matrix/filler interface.

In regard to PLA, several types of natural fibers have been used to produce biocomposites [13,14,15,16,17,18]. The effect of the processing conditions and, in some cases, surface chemical modification of the fibers on mechanical, rheological, and thermal properties of PLA/fibers biocomposites has been widely investigated [13,14,15,16,17,18].

Organic wastes have sometimes been utilized as reinforcements or additives for various polymers, examples being charcoal and lignin [19]. By considering that significant amounts of organic wastes from industry and agriculture are unutilized, it follows that the use of organic residue materials in biocomposites can represent an ecologically friendly method to produce materials for different applications. This can also allow the reduction of the cost of the products.

Potato wastes are biomasses rich in starch and lignocellulosic constituents. After extraction of the starch, the potato pulp accumulates in high amounts—approximately 0.75 tons of pulp arises per ton of purified starch. Within the European Union, about 140,000 tons of dried potato pulp are produced annually in the starch industry [20]. The original potato pulp contains water up to about 90%, but de-watering processes generally results in an increase in the dry matter up to about 90 wt%. Dried potato pulp, which consists mainly of lignocellulosic fibers, starch, and proteins, can be used as filler or for a reinforcement action on bio-based polymers. The cost of this raw material is low, which makes potato pulp even more interesting for industrial utilization [21].

In the present work, biocomposites made of poly(lactic acid) and potato pulp powder (PPP) have been produced by extrusion followed by injection molding and characterized in terms of process ability and thermal, mechanical, and rheological properties. It is worth pointing out that the composition of the potato pulp powder is expected to depend on the geographical origin of the agricultural product and on the climatic and harvesting conditions as well as on the industrial processing methods. For this reason, the present study provides only a general description of the properties of these PLA-based biocomposites, which could slightly change with varying the original materials. In order to make the processing of PLA with the potato pulp powder easier, a plasticizer, acetyl tributyl citrate (ATBC), was used. ATBC is an efficient plasticizer of PLA [22]. It is derived from naturally occurring citric acid, non-toxic, and accepted for food-contact [23]. In addition, calcium carbonate (CaCO_3_) was used in low percentages as an inert filler to facilitate the removal of the injection-molded specimens from the mold. The thermal, mechanical, and rheological properties of the PLA-based matrix were investigated as preliminary step in order to better quantify the influence of the reinforcing fibers on the material properties.

The PLA-based samples investigated in the present study, with the relative composition, are listed in Table 1. All the samples were processed as described in Section 3.

## 2. Results and Discussion

### 2.1. Thermogravimetric Analysis of the Potato Pulp Powder, the PLA-Based Matrix, and the Biocomposite [PLA (85 wt%) + ATBC (10 wt%) + CaCO_3_ (5 wt%)] + PPP (20 wt%)]

The main drawback of the use of natural fibers as reinforcement or fillers in biocomposites is the relative low processing temperature required due to the thermal degradation that occurs at high temperatures which can irreversibly damage them. As all the components of the potato pulp can be subjected to thermal degradation during composite processing, it is of practical significance to investigate the thermal decomposition process of the potato pulp powder in order to estimate the temperature range in which the biocomposites can be processed.

**Figure 1:**
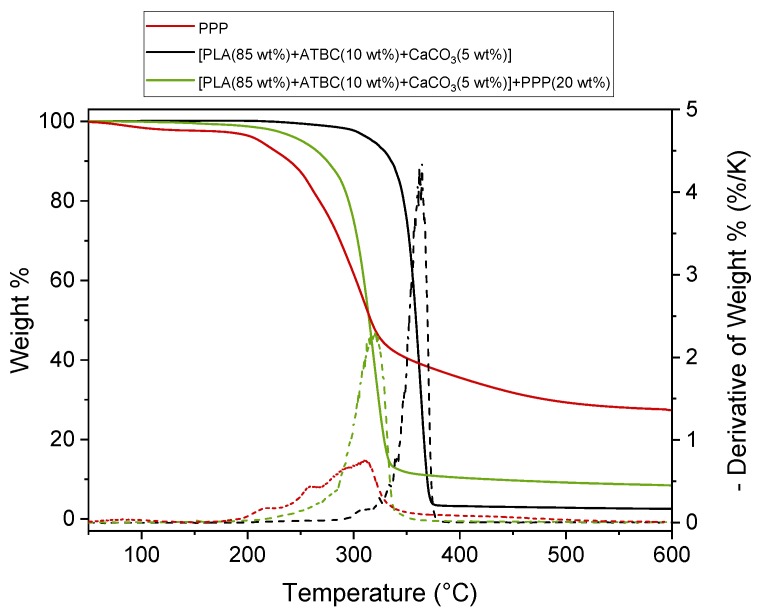
Thermogravimetric curves of the potato pulp powder (PPP), the poly(lactic acid) (PLA)-based matrix andbiocomposite [PLA (85 wt%) + acetyl tributyl citrate (ATBC; 10 wt%) + CaCO_3_ (5 wt%)] + potato pulp powder (PPP; 20 wt%) at 10 K/min under nitrogen flow. The dotted lines are the derivatives of the weight % curves.

The thermal stability of the potato pulp powder was determined by means of the thermogravimetric analysis under nitrogen flow, because the contact of the material with air is reduced in the extruder and molder. Figure 1 shows the thermogravimetric curve of the potato pulp powder, which reports the change in weight according to a fixed temperature program. The initial weight loss detected at temperatures lower than 150 °C is due to water vaporization. The water content in PPP is approximately 3 wt%. The weight residue that is observed at high temperature is due to the carbon deposit that remains in the presence of an inert atmosphere. The thermal degradation of natural fibers generally has multiple processes due to the different components [24,25]. Degradation of hemicellulose occurs mainly in the temperature range of 200–350 °C with the maximum mass loss rate at around 300 °C, whereas cellulose pyrolysis takes places at higher temperatures, between 250 and 400 °C. At high temperatures, the solid residue of pure hemicellulose and cellulose is about 20%. Lignin is the most difficult component to decompose; its degradation takes place slowly and continuously from about 250 °C, with a high solid residue that remains at high temperatures (around 50%). Starch degradation extends from approximately 300 to 350 °C, and its solid residue at 500 °C is approximately 20% [26]. Degradation of proteins also occurs in a similar temperature range, between 200 and 400 °C, with a maximum loss rate at around 300 °C and 20% residue at high temperatures [27]. From Figure 1, the potato pulp powder appears stable up to about 180 °C. This thermal stability assures that the potato pulp powder does not undergo substantial degradation during the processing of the PLA biocomposites at 180 °C, being that the residence time at this temperature is no longer than 1.5 min. It can therefore be concluded that the potato pulp can be confidently used for the production of PLA-based biocomposites.

Figure 1 shows also the thermogravimetric curves of the PLA-based matrix and biocomposite with 20 wt% of PPP. Both these thermal degradations occur in a single step in a narrow temperature range. The initial degradation temperature of the PLA-based matrix is located at about 300 °C, whereas the maximum degradation rate is centered at about 365 °C, which is in excellent agreement with previous studies [28,29,30] in which the independence of the PLA thermal stability on the molar mass was also demonstrated [28]. The biocomposite with 20 wt% of potato pulp powder starts to degrade at about 180 °C, whereas the maximum degradation rate shifts to around 320 °C, thus approaching the thermal degradation of plain potato pulp powder. As expected, the residue of the biocomposite at 600 °C is higher compared to that of the PLA based matrix due to the presence of the lignocellulosic residue. In conclusion, Figure 1 reveals that the processing of the biocomposites at 180 °C does not substantially affect the potato pulp powder structure because the degradation of the biocomposite starts at the same temperature of the original unprocessed potato pulp powder.

### 2.2. Scanning Electron Microscopy of the Potato Pulp Powder

**Figure 2:**
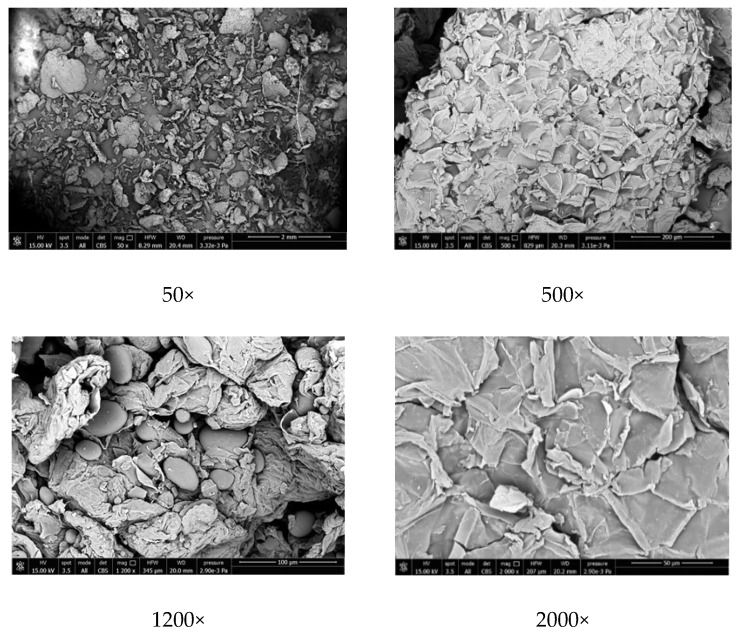
Scanning electron microscopy (SEM) images of the potato pulp powder (PPP) at the magnifications indicated.

The morphologies of the potato pulp powder were investigated with scanning electron microscopy (SEM), and SEM images are shown in Figure 2. A homogeneous distribution of pulp fragments, which appear as small aggregates, can be observed. The aggregates are relatively large (200 µm and more). The round-shaped particles detected at 1200× magnification in the potato pulp powder are either starch or pectin because they disappear after treatment with amylase and pectinase. 

### 2.3. Thermal, Mechanical, and Rheological Properties of the PLA-Based Matrix

The thermal, mechanical, and rheological properties of the PLA-based matrix were investigated as a preliminary step in order to better quantify the influence of the reinforcing fibers on the material properties.

**Figure 3:**
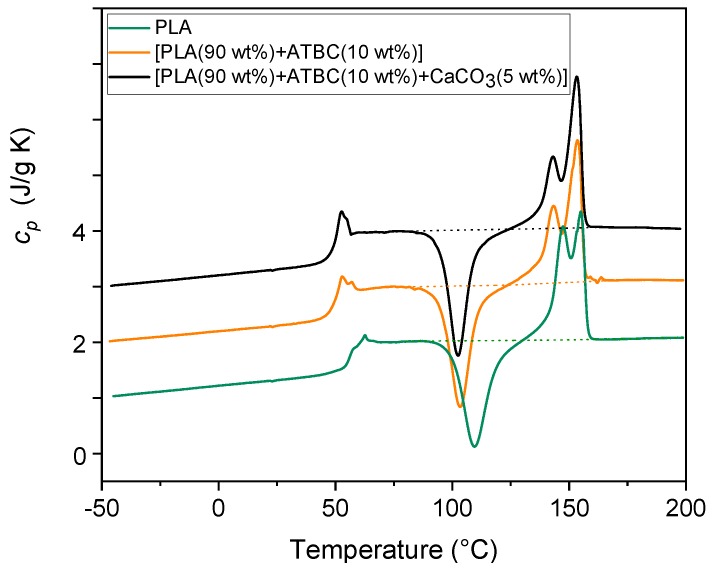
Specific heat capacity (*c_p_*) of PLA and PLA mixed with (1) ATBC and (2) ATBC and the mineral filler CaCO_3_ at the concentrations indicated. The curves were obtained upon heating at 10 K/min after previous fast cooling to −50 °C. The dotted lines are the baselines used for the calculation of the Δ*h_c_* and Δ*h_m_* values. The ordinate values refer only to the bottom curve. All the other curves are shifted vertically for the sake of clearness.

The specific heat capacity (*c_p_*) curves of PLA and PLA mixed with (1) the plasticizer ATBC and (2) with ATBC and the mineral filler CaCO_3_, measured at 10 °C/min, are shown in Figure 3. As described in Section 3, the samples were injection-molded for 1 min at 90 °C. The glass transition, which occurs in the proximity of 60 °C, which is in agreement with literature data [31], is overlapped by an enthalpy recovery peak due to the permanence of the samples at room temperatures for one day. Before the melting endotherm, all the curves displayed an intense cold crystallization peak located approximately in the interval between 90 and 130 °C. In the temperature range between 100 and 120 °C, both the crystalline α′- and α-forms grew [32,33]. Thus the melting behavior that extends from approximately 130 to 160 °C results from the fusion of both the α- and the α’-crystals. At the heating rate of 10 K/min, the α’-crystals transform into the more ordered α-phase via melting and almost simultaneous recrystallization, with the result that a multiple melting behavior, independent of the molar mass, is commonly observed [34,35]. Reorganization and recrystallization events overlap the entire fusion process, which generally takes place in semi-crystalline polymers at a relatively low heating rate [36,37].

Table 2 lists the *T_g_* values together with the enthalpy of cold crystallization (Δ*h_c_*) and the enthalpy of fusion (Δ*h_m_*) of the samples investigated in this section, calculated from the *c_p_* curves shown in Figure 3. The Δ*h_c_* and Δ*h_m_* values collected in Table 2 are normalized to the PLA content. From these experimental values, an estimation of the crystalline weight fraction growing during the cold crystallization process (*w_Cc_*) and disappearing during the melting process (*w_Cm_*) was obtained by dividing Δ*h_c_* and Δ*h_m_* by the enthalpy of fusion of 100% crystalline PLA phase (Δ*h_m_*°) at the crystallization and melting peak temperatures, respectively. As both the α′- and α-forms grow during cold crystallization and disappear during the melting process, the average values between the enthalpy of fusion of the α′- and α-forms were utilized [38], i.e., Δ*h_m_*° = 101 J/g for the cold crystallization centered at about 110 °C, Δ*h_m_*° = 96 J/g for the cold crystallization centered at about 100 °C, and Δ*h_m_*° = 119 J/g for the melting process centered approximately at 150 °C. The *w_Cc_* and *w_Cm_* values listed in Table 2 reveal that the PLA and the PLA samples with the addition of (1) only the plasticizer ATBC and (2) the plasticizer ATBC and the mineral filler CaCO_3_, after processing for 1 min at 90 °C, are completely amorphous. This can be explained by considering the short times that characterize a typical injection molding and the slow crystallization kinetics of PLA [39]. In the presence of ATBC, the crystallinity that grows during the heating run is higher with respect to PLA, which can be ascribed to the higher mobility achieved by the PLA chains. The plasticizing effect of ATBC on PLA is evidenced by the decrease in the *T_g_* value, the reduced peak temperature of the cold crystallization, and the higher crystallinity that develops upon heating, whereas the inertia of CaCO_3_ towards the PLA phase transitions is proven by the unchanged thermal parameters.

**Figure 4:**
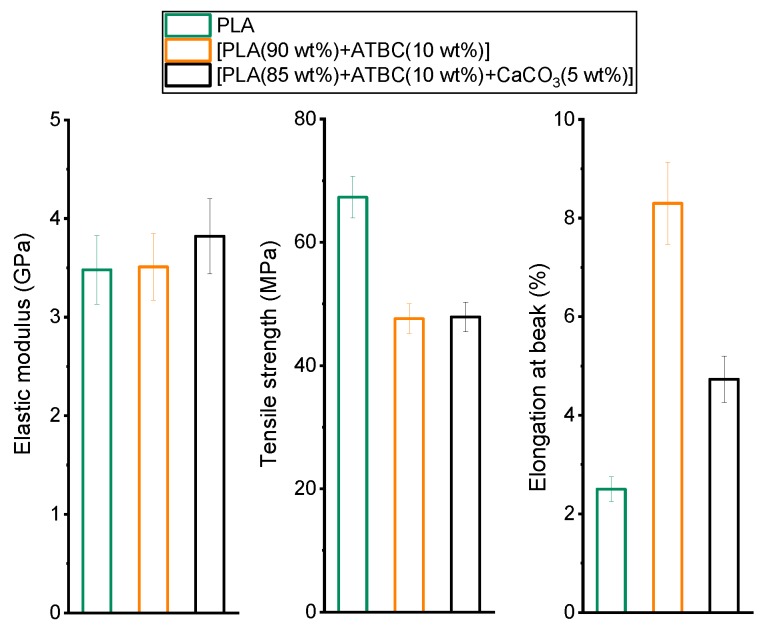
Elastic modulus, tensile strength and elongation at break of PLA and PLA mixed with (1) ATBC and (2) ATBC and the mineral filler CaCO_3_ at the concentrations indicated.

The mechanical properties of PLA and of PLA after processing in the presence of (1) the plasticizer ATBC and (2) the plasticizer ATBC with the mineral filler CaCO_3_ are summarized in Figure 4. PLA is a quite brittle polymer with a high elastic modulus and a high tensile strength. The addition of the plasticizer ATBC, as expected, modifies the mechanical properties: The elastic modulus does not substantially change, the tensile strength decreases, and conversely, the elongation at break greatly increases. In the presence of the plasticizer ATBC, the intermolecular forces between the PLA chains decrease, the mobility of the polymeric chains enhances, and an increase in flexibility and ductility is produced. A similar behavior has been reported for other PLA plasticized systems [40,41].

The addition of the mineral filler CaCO_3_ produces a slight increase in the elastic modulus and a strong decrease in the elongation at break, whereas it has negligible effect on the tensile strength. This behavior is in agreement with literature data. The modulus increase is the result of the reinforcement effect of CaCO_3_ particles. However, if the mineral filler is present in small percentages, the influence on the elastic modulus is small because it can be assumed that the perturbed polymer fraction around each mineral particle is low compared with the unperturbed one [42,43]. The tensile strength remains unaffected by the presence of the mineral filler, which means that the mobility of the polymer chains is not influenced by the small percentage of CaCO_3_ as also proven by the constant *T_g_* value. Conversely, the elongation at break strongly decreases because the presence of micro-sized mineral particles modifies the fracture mechanism and hinders the elongation of the material. Mineral particles generally act as stress concentrators capable of initiating cracking and favoring specific fracture mechanisms.

The study of rheological properties of PLA is crucial for gaining a fundamental understanding of the processability of PLA materials, especially for injection molding and extrusion processes. PLA behaves like a pseudo-plastic, a non-Newtonian fluid, and a typical shear thinning fluid in which, at high shear rates, the macromolecules orient and decrease the entanglement number [44,45]. 

**Figure 5:**
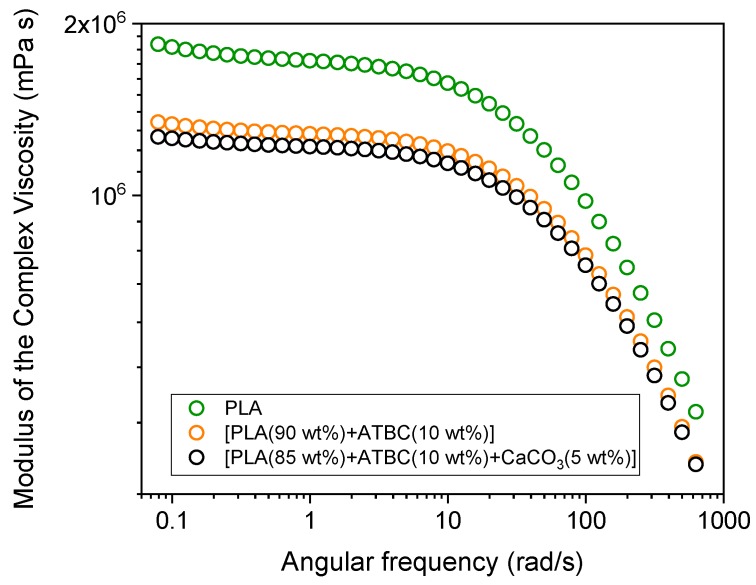
Modulus of the complex viscosity vs. angular frequency at 175 °C for PLA and PLA with the addition of (1) the plasticizer ATBC, and (2) the plasticizer ATBC and the mineral filler CaCO_3_.

Figure 5 shows the dependence of the modulus of the complex viscosity on the deformation frequency for PLA and PLA with the addition of (1) the plasticizer ATBC, and (2) the plasticizer ATBC and the mineral filler CaCO_3_. The rheological measurements were performed from low to high frequencies. All the curves indicate a decrease in complex viscosity with increasing rotational frequency. In the terminal zone, which defines the zero-shear viscosity η_o_, a small decrease in viscosity is observed, probably due an original entangled structure not completely destroyed before the beginning of the rheological test. The η_o_ value for PLA at 175 °C is about 1.8 × 10^6^ mPa·s. This value is in the range typical of commercial polymers (*M_w_* = 140–160 kg/mol), i.e., 10^5^–10^7^ mPa·s [46]. The incorporation of ATBC reduces the viscosity due to its plasticizing effect, which leads to a decrease in the intermolecular forces and an increase in the mobility of the polymeric chains [47,48]. The addition of CaCO_3_ causes a further small reduction of viscosity, which is in excellent agreement with literature data [49]. A low CaCO_3_ content is insufficient to form a filler network. As a consequence, the incorporation of the mineral filler weakens the interaction between PLA chains segments, thus producing a viscosity reduction.

### 2.4. Thermal, Mechanical and Rheological Properties of the PLA-Based Biocomposites

The thermal, mechanical, and rheological properties of the biocomposites of PLA with potato pulp powder were investigated in order to quantify how the addition of these fibers modifies the structure of the polymeric material. These data together provide information on the possible miscibility and compatibility between PLA and potato pulp. The PLA-based biocomposites with potato pulp were studied by analyzing the effect of potato pulp concentration. 

**Figure 6:**
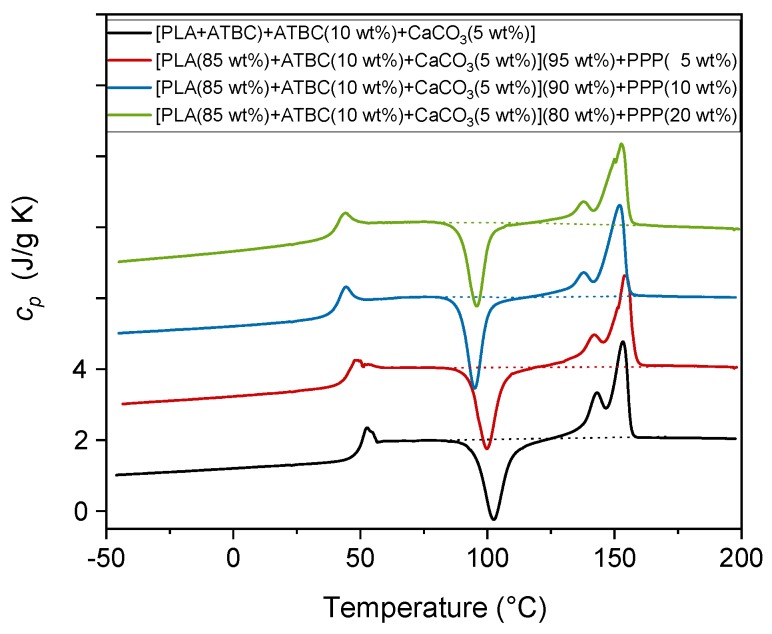
Specific heat capacity (*c_p_*) of the PLA-based matrix and biocomposites indicated in the legend. The curves were obtained upon heating at 10 K/min after previously fast cooling to -50 °C. The dotted lines are the baselines used for the calculation of the Δ*h_c_* and Δ*h_m_* values. The ordinate values refer only to the bottom curve. All the other curves are shifted vertically for the sake of clarity.

The specific heat capacity (*c_p_*) curves of the [PLA (85 wt%) + ATBC (10 wt%) + CaCO_3_ (5 wt%)] matrix with the addition of an increasing percentage of PPP (5 wt%, 10 wt%, and 20 wt%), measured at 10 K/min, are shown in Figure 6. 

Table 3 lists the *T_g_* values together with the enthalpy of cold crystallization (Δ*h_c_*) and the enthalpy of fusion (Δ*h_m_*) of the samples investigated in this section as calculated from the *c_p_* curves shown in Figure 6. The addition of PPP affects the glass transition temperature, which decreases with increasing the PPP percentage. This reveals that the polymer chain mobility increases after addition of the potato pulp powder. This can be ascribed to the development of weak intermolecular interactions between PLA and the potato pulp particles, which could cause the formation of outspread free volume at the matrix/fiber interface.

The peak temperature of the cold crystallization process decreases in the biocomposites, proving that PPP plays a nucleating role during the heating run, which accelerates the PLA cold crystallization process. This a general behavior of the natural fibers, as also found in other PLA/natural fibers composites [15,50], which has been explained as due to the formation of a transcrystalline layer around the fibers. The occurrence of transcrystallinity was reported for a large combination of semi-crystalline thermoplastic matrices and fibers [51,52]. For biocomposites containing 10 and 20 wt% of PPP, cold crystallization occurs almost completely at temperatures lower than 100 °C, which means that only the α′-form develops [32,33]. At the heating rate of 10 K/min, however, these α’-crystals are expected to transform into the more ordered α-phase [34]. For biocomposites with 10 and 20 wt% of PPP, *w_Cc_* was calculated by dividing the measured Δ*h_c_* by the enthalpy of fusion of the α′-form at about 95 °C (Δ*h_m_*° = 81 J/g), whereas *w_Cm_* was obtained from the ratio between Δ*h_m_* and the enthalpy of fusion of the α′-form at about 150 °C (Δ*h_m_*° = 108 J/g) [38], as the enthalpy of crystallization and the enthalpy of fusion of the α-crystals cancel out. For the biocomposite with 5 wt% of PPP, for which cold crystallization extends up to above 110 °C, the average values between the enthalpy of fusion of the α′- and α-forms were utilized, i.e., Δ*h_m_*° = 96 J/g for the cold crystallization centered at about 100 °C, and Δ*h_m_*° = 119 J/g for the melting process centered approximately at 150 °C [38].

The *w_Cc_* and *w_Cm_* values listed in Table 3, as derived from the measured Δ*h_c_* and Δ*h_m_* values, respectively, reveal that the PLA-based biocomposites containing PPP, processed for 1 min at 90 °C, are completely amorphous. Despite the nucleating action of PPP, which leads to a progressively and slightly higher crystallinity to develop upon heating, the residence time of the materials in the mold is too short to allow the crystallization of PLA.

**Figure 7:**
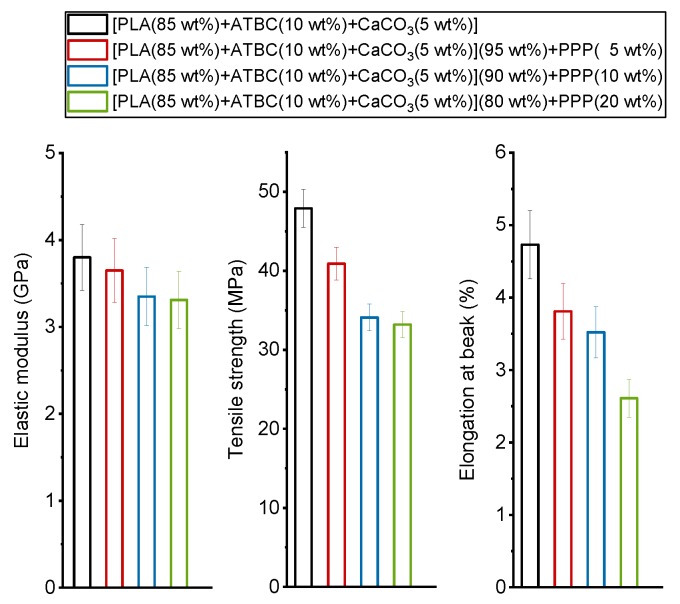
Elastic modulus, tensile strength, and elongation at break of the PLA-based matrix and biocomposites indicated in the legend.

The mechanical properties of the [PLA (85 wt%) + ATBC (10 wt%) + CaCO3 (5 wt%)] matrix and the biocomposites with potato pulp powder are summarized in Figure 7.

As a general rule, the modulus of a composite depends on the modulus of both the matrix and the reinforcement and filler, and the modulus of the natural fibers is generally higher than the modulus of the polymeric matrices [12]. Figure 7 reveals that the elastic modulus decreases by increasing the PPP percentage, which could mean that PPP acts for PLA matrix as filler and not as reinforcement, or it could be ascribed to PLA degradation during the processing step induced by fiber moisture [53]; although, both PLA and the PPP powder were previously dried for a long time, and the water content of the potato pulp powder was about 3 wt% (Figure 1). The number-average molar mass (*M_n_*) and the mass-average molar mass (*M_w_*) of PLA of both the matrix and the biocomposite with 20 wt% of potato pulp powder processed under identical conditions were measured by size exclusion chromatography (SEC). For the PLA-based matrix, *M_n_* = 92,000 g/mol and *M_w_* = 170,000 g/mol, whereas for the biocomposite [PLA (85 wt%) + ATBC (10 wt%) + CaCO_3_ (5 wt%)] (80 wt%) + PPP (20 wt%), *M_n_* = 70,000 g/mol and *M_w_* = 130,000 g/mol were obtained. The observed decrease of about 20% in the PLA molar mass can thus be ascribed to the PLA degradation occurring during processing in the presence of the moisture introduced by the potato pulp powder. This could explain the loss in the mechanical properties. However, for *M_w_* higher than about 100,000 g/mol, the mechanical properties of PLA were not strongly influenced by the molar mass [54]. Thus, besides the possible effect of PLA degradation induced by the fiber moisture, the reduction in the elastic modulus of the PLA-based biocomposites with potato pulp powder has to be ascribed also to poor adhesion between the matrix and the filler. Potato pulp powder is composed mainly of lignocellulosic fibers and starch, which are highly hydrophilic, so poor interactions are expected at the interface with the less hydrophilic PLA. Poor adhesion between the potato pulp powder and the polymeric matrix is also evidenced by the tensile strength, which decreases with PPP percentage. The elongation at break is reduced drastically because the dispersed filler particles act as stress concentrators at the polymer/filler interface by inhibiting the deformation, which leads to reduced ductility of the material.

**Figure 8:**
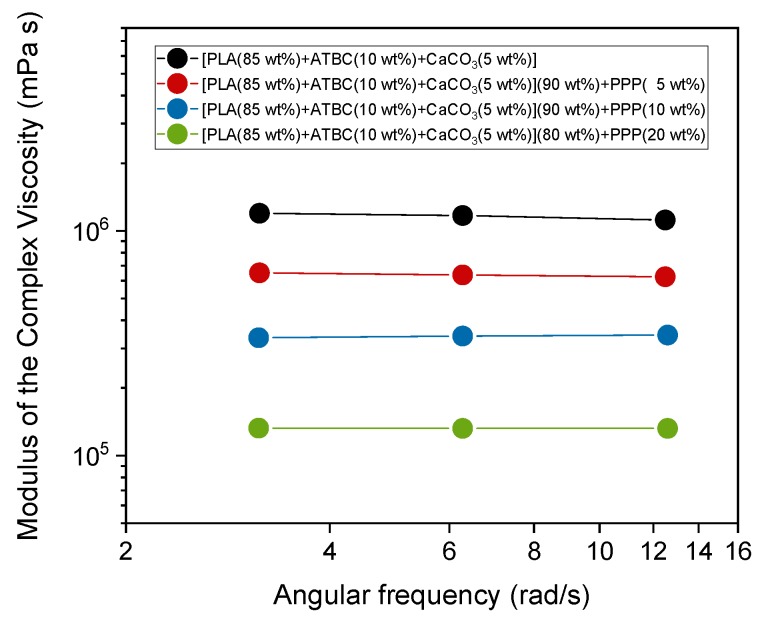
Modulus of the complex viscosity vs. angular frequency at 175 °C for PLA-based matrix and biocomposites indicated in the legend.

Figure 8 shows the dependence of the modulus of the complex viscosity on the deformation frequency at 175 °C for the PLA-based biocomposites containing potato pulp powder. The experiments were performed from high to low frequencies in a narrow deformation frequency range outside but not far from the Newtonian plateau in order to considerably reduce the measurement times, and consequently, fiber degradation. This also allows the comparison between the viscoelastic behaviors of the different biocomposites. Figure 8 indicates that the viscosity of the biocomposites is smaller than that of the polymeric matrix and decreases with an increase in PPP percentage. For *M_w_* > 100,000 g/mol, the zero shear viscosity η_o_, referring to the Newtonian plateau, can be described by the relationship: log η_o_ = log K + 3.4 log *M_w_* [55,56]. Assuming for the PLA-based matrix that log η_o_ ≈ 6.1 (Figure 5), the molar mass reduction detected for the [PLA (85 wt%) + ATBC (10 wt%) + CaCO_3_ (5 wt%)] (80 wt%) + PPP (20 wt%) biocomposite should produce a decrease in log η_o_ of about 7%. The decrease in the modulus of the complex viscosity for the biocomposite with 20 wt% of PPP is actually about 16% in the investigated deformation frequency range, which is close to the Newtonian plateau. This finding further confirms that the presence of free volume at the polymer/fiber interfaces due to poor adhesion between potato pulp powder and PLA reduces the viscosity in the biocomposites and favors the flow of the PLA chains.

### 2.5. Morphological Properties of the PLA-Based Biocomposites

The morphology of fracture surfaces of the PLA-based biocomposites with PPP from dog-bone specimens was studied by scanning electron microscopy in order to investigate the dispersion of the potato pulp particles and the compatibility between the matrices and the fibers. 

**Figure 9:**
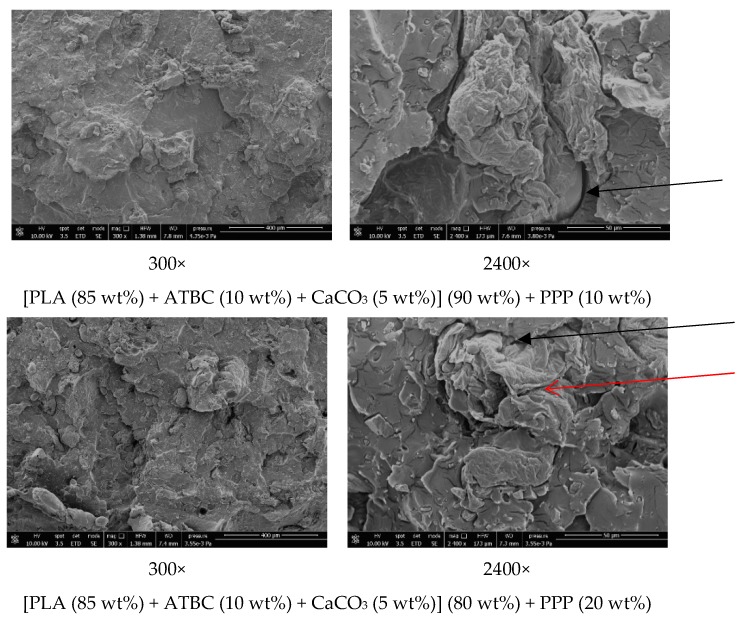
SEM images of the [PLA (85 wt%) + ATBC (10 wt%) + CaCO_3_ (5 wt%)] (90 wt%) + PPP (10 wt%) and [PLA (85 wt%) + ATBC (10 wt%) + CaCO_3_ (5 wt%)] (80 wt%) + PPP (20 wt%) biocomposite at the indicated magnification.

Figure 9 shows that the potato pulp particles are well dispersed within the matrix and their distribution is uniform, which means that they were satisfactorily separated during the extrusion process. The micrographs clearly show that the interfacial adhesion between the PLA-based matrix and the potato pulp powder is quite poor because a gap is well evident between the polymeric matrix and the potato pulp particles as indicated by the black arrows. Poor adhesion leads to brittle materials as also proven by the fiber pullout indicated by the red arrow, which illustrates how, in these biocomposites, failure occurs at the matrix/fiber interface. The finding is in agreement with the decrease in the tensile strength discussed above (Figure 7).

## 3. Materials and Methods 

### 3.1. Materials

Poly(lactic acid) (PLA), derived from natural resources, was obtained from 2003D NatureWorks (Minnetonka, MN, USA). The product grade was suitable for thermoforming and extrusion processes and contained 3% d-lactic acid units (melt flow index (MFI): 6 g/10 min (210 °C, 2.16 kg); nominal average molar mass: 200,000 g/mol). 

The plasticizer acetyl tributyl citrate (ATBC) was purchased from Sigma Aldrich S.R.L (Milan, Italy).

The calcium carbonate (CaCO_3_) OMYACARB^®^ was an inert filler supplied by the company OMYA (Oftringen, Switzerland). The powder had fine granulometry with particle size distribution centered at 12 μm. 

Dried potato pulp powder (PPP) was produced by the company SüdStärke (Schrobenhausen, Germany). The moisture content was about 3 wt%, and the composition of the dry matter was as follows: proteins 7 wt%, starch 25 wt%, cellulose 16 wt%, hemicellulose 7 wt%, lignin 20 wt%, pectin 17 wt%, and ash 5 wt%.

### 3.2. Composite Preparation

Biocomposites of PLA with potato pulp powder were prepared by adding the filler PPP in different percentages to the polymeric matrix constituted by the bio-based PLA (with a concentration of 85 wt%), the plasticizer ATBC (with a concentration of 10 wt%), and CaCO_3_ (with a concentration of 5 wt%). For comparison, pure PLA and PLA mixed only with the plasticizer ATBC were also processed in the same way.

Before processing, the PLA and potato pulp powder were dried at a temperature of 60 °C for at least 24 h. The PLA-based matrix and biocomposites were prepared by using a MiniLab II HAAKE Rheomex CTW 5—a co-rotating conical twin-screw extruder. The molten materials were transferred from the mini extruder through a preheated cylinder to a mini injection molder (Thermo Scientific HAAKE MiniJet II), which allows the preparation of dog-bone tensile bar specimens to be used for thermal, mechanical, and rheological characterization. The dimensions of the dog-bone tensile bars were as follows: Width in the larger section—10 mm, width in the narrow section—4.8 mm, thickness—1.35 mm, and length—90 mm. The extruder operating conditions adopted for all the formulations are reported in Table 4.

After preparation, all the samples were stored in a desiccator and analyzed the day after in order to avoid physical ageing effects on the physical properties investigated.

### 3.3. Composite Characterization

The thermal stability of the potato pulp powder and selected samples was investigated by thermogravimetric analysis (TGA) carried out on about 10 mg of sample by using a SII TG/DTA 7200 EXSTAR Seiko (Minato, Tokyo, Japan) under nitrogen flow (200 mL/min) and at a heating speed of 10 K/min from 50 to 600 °C. 

Number-average molar mass (*M_n_*) and weight-average molar mass (*M_w_*) were determined using size exclusion chromatography (SEC) with the Agilent Technologies 1200 Series (Santa Clara, CA, USA) and calculated with the Agilent ChemStation Software. The instrument was equipped with an Agilent degasser, an isocratic HPLC pump, two PLgel 5 μm MiniMIX-D columns conditioned at 35 °C, and an Agilent refractive index (RI) detector. The mobile phase was chloroform (CHCl_3_) at a flow rate of 0.3 mL/min. The system was calibrated with polystyrene standards in a range from 500 to 3 × 10^5^ g/mol. Samples were dissolved in CHCl_3_ (2 mg/mL) and filtered through a 0.20 μm syringe filter before analysis. 

The morphology of the PPP powder was investigated by scanning electron microscopy (SEM) with an FEI-Quanta 450 ESEM instrument (ThermoFisher, Waltham, MA, USA). The micrographs of samples fractured with liquid nitrogen and etched with gold were collected. Backscattered electrons generated the images, the resolution for which was provided by beam deceleration with a landing energy of 2 kV.

Differential scanning calorimetry (DSC) measurements were performed with a Calorimeter DSC 8500 Perkin Elmer (Waltham, MA, USA) equipped with an IntraCooler III as a refrigerating system. The instrument was calibrated to temperature with high purity standards (indium, naphthalene, cyclohexane) according to the procedure for standard DSC [57]. Energy calibration was performed with indium. Dry nitrogen was used as a purge gas at a rate of 30 mL/min. The as prepared samples were analyzed from −50 to 200 °C at the heating rate of 10 K/min and with a single thermal scan. 

Tensile tests on the samples prepared with the injection molder were performed at room temperature at a crosshead speed of 10 mm/min and by means of an INSTRON 5500 R universal testing machine (Instron Corp., Canton, MA, USA) equipped with a 10 kN load cell and interfaced with a computer running the Testworks 4.0 software (MTS Systems Corporation, Eden Prairie, MN, USA). At least five specimens were tested for each sample in accordance to ASTM D 638, and the average values were reported.

Oscillatory shear measurements were performed by means of a Rheometer Anton Paar MCR 302 (Graz, Austria) equipped with parallel plates of 25 mm diameter under nitrogen flow to minimize oxidation and to maintain a dry environment. Frequency sweep experiments were performed at 175 °C at a fixed strain (3%) with a gap of 1 mm, in the linear regime, in order to measure the modulus of the complex viscosity. For the analysis of the PLA-based matrix, the angular frequencies were swept from 0.068 to 628 rad/s with five points per decade. For the analysis of the biocomposites, a narrower angular frequency was covered to reduce the time measurement and avoid degradation of the potato pulp.

## 4. Conclusions

Potato pulp powder, which is an organic waste from the production and extraction of starch, can be suitable for processing in the melt with the bio-based and biodegradable PLA, presenting no main problems in processing. Due to the low aspect ratio of the potato pulp powder, there is no reinforcing effect on the polymeric matrix, but there is a moderate loss in mechanical properties. The potato pulp power utilized to produce PLA-based biocomposites acts as a filler and not as a reinforcement for the polymeric matrix. The loss in mechanical properties is, however, not dramatically high, because the elastic modulus decreases by only 13% when the PPP content is 20 wt%. The adhesion between the potato pulp powder and the polymeric matrices is poor, as attested by the tensile strength, which appears progressively smaller for the biocomposites with respect to the PLA matrix. The ductility of the biocomposites is also smaller with respect to that of the PLA matrix because the dispersed potato pulp particles act as stress concentrators and promote microcrack formation. Conversely, the lower viscosity of the biocomposites containing PPP is a factor that favors the processing and the molding of these materials. 

Notwithstanding, the present PLA-based biocomposites still present properties valuable for practical applications and still meet the technical requirements indicated for rigid packaging production. In addition, the presence of natural fibers promotes and accelerates the biodegradation of the biocomposite material [58].

Finally, the incorporation of about 20 wt% of potato pulp powder, no-food competition biomass, to PLA offers the possibility to markedly reduce the cost of the final products when considering the relatively high cost of PLA. This opens an innovative option for the valorization of an abundant agro-food biomass such as potato pulp and thus meeting circular economy expectations. 

## Figures and Tables

**Table 1:** Composition of the poly(lactic acid) (PLA)-based matrix and biocomposites.

	Potato Pulp
PLA (100%)	-
[PLA (90 wt%) + ATBC (10 wt%)]	-
[PLA (85 wt%) + ATBC (10 wt%) + CaCO_3_ (5 wt%)]	-
[PLA (85 wt%) + ATBC (10 wt%) + CaCO_3_ (5 wt%)] (95 wt%)	PPP (5 wt%)
[PLA (85 wt%) + ATBC (10 wt%) + CaCO_3_ (5 wt%)] (90 wt%)	PPP (10 wt%)
[PLA (85 wt%) + ATBC (10 wt%) + CaCO_3_ (5 wt%)] (80 wt%)	PPP (20 wt%)

**Table 2:** Glass transition temperatures (*T_g_*), enthalpy of cold crystallization (*Δh_c_*), enthalpy of fusion (*Δh_m_*), and crystalline weight fraction growing during cold crystallization (*w_Cc_*) and disappearing during fusion (*w_Cm_*) for PLA and PLA mixed with (1) ATBC and (2) ATBC and the mineral filler CaCO_3_ (estimated errors: ± 0.5 °C for *T_g_*; ± 1 J/g for *Δh_c_* and *Δh_m_*, and ± 0.02 for *w_Cc_* and *w_Cm_*).

	*T_g_* (°C)	Δ*h_c_* (J/g)	*w_Cc_*	*Δh_m_* (J/g)	*w_Cm_*
PLA	58	24	0.24	28	0.24
[PLA (90 wt%) + ATBC (10 wt%)]	50	27	0.28	33	0.28
[PLA (85 wt%) + ATBC (10 wt%) + CaCO_3_ (5 wt%)]	50	27	0.28	33	0.28

**Table 3:** Glass transition temperatures (*T_g_*), enthalpy of cold crystallization (Δ*h_c_*), enthalpy of fusion (Δ*h_m_*), and crystalline weight fraction growing during cold crystallization (*w_Cc_*) and disappearing during fusion (*w_Cm_*) for the PLA-based matrix and biocomposites (estimated errors: ± 0.5 °C for *T_g_*; ± 1 J/g for Δ*h_c_* and Δ*h_m_*, and ± 0.02 for *w_Cc_* and *w_Cm_*).

	*T_g_* (°C)	Δ*h_c_* (J/g)	*w_Cc_*	Δ*h_m_* (J/g)	*w_Cm_*
[PLA (85 wt%) + ATBC (10 wt%) + CaCO_3_ (5 wt%)]	50	27	0.28	33	0.28
[PLA (85 wt%) + ATBC (10 wt%) + CaCO_3_ (5 wt%)] (95 wt%) + PPP (5 wt%)	45	26	0.27	34	0.28
[PLA (85 wt%) + ATBC (10 wt%) + CaCO_3_ (5 wt%)] (90 wt%)+PPP (10 wt%)	40	26	0.32	35	0.32
[PLA (85 wt%) + ATBC (10 wt%) + CaCO_3_ (5 wt%)] (80 wt%)+PPP (20 wt%)	40	28	0.34	37	0.34

**Table 4:** Operating condition use for the extrusion and injection molding process.

Extrusion Temperature (˚C)	Screw Speed (rpm)	Cycle Time (s)	Injection Temperature (˚C)	Injection Pressure (bar)	Molding Time (s)	Mold Temperature (˚C)
180	100	90	180	500	60	90
